# A preliminary investigation into the impact of soft tissue augmentation-based periodontal phenotype modification therapy for patients exhibiting class III decompensation

**DOI:** 10.1186/s12903-024-04630-x

**Published:** 2024-08-02

**Authors:** Mengdi Li, ZhiXu Liu, Xiao Yang, Min Zhu, Jing Ni

**Affiliations:** 1 Department of Periodontology, Shanghai Ninth People’s Hospital, Shanghai Jiao Tong University School of Medicine; College of Stomatology, Shanghai Jiao Tong University; National Center for Stomatology; National Clinical Research Center for Oral Diseases; Shanghai Key Laboratory of Stomatology; Shanghai Research Institute of Stomatology, 639 Zhizao Ju Road, Shanghai, 200011 China; 2 Department of Oral & Craniomaxillofacial Surgery, Center of Craniofacial Orthodontics, Shanghai Ninth People’s Hospital, Shanghai Jiao Tong University School of Medicine; Shanghai Key Laboratory of Stomatology & Shanghai Research Institute of Stomatology; National Clinical Research Center of Stomatology, Shanghai, China

**Keywords:** Skeletal angle class III malocclusion, Gingival recession, Periodontal phenotype modification, Soft tissue augmentation, Soft tissue grafting

## Abstract

**Background:**

Patients with skeletal angle Class III malocclusion usually have inadequate hard and soft tissue volume at the mandibular anterior teeth. The labial proclination at the teeth may lead to gingival recession. The purpose of this study was to explore whether periodontal phenotype modification therapy with soft tissue augmentation (PhMT-s) can prevent gingival recession in these patients.

**Methods:**

Four patients with skeletal Class III malocclusion and a thin periodontal phenotype underwent surgical-orthodontic treatment. Prior to tooth movement, they underwent a minimally invasive vestibular incision with subperiosteal tunnel access combined with autogenous connective tissue grafts for periodontal phenotype modification with soft tissue augmentation (PhMT-s). The labial gingival thickness of the anterior mandibular teeth was measured at three distinct levels: at the cementoenamel junction (GT0), 3 mm apical to the CEJ (GT3), and 6 mm apical to the CEJ (GT6). These measurements were taken at baseline, three months following PhMT-s, and after tooth decompensation. Additionally, a biopsy sample was obtained from the PhMT-s site of one patient. All sections were subsequently stained using hematoxylin and eosin, Masson trichrome, Sirius Red, and immunohistochemistry.

**Results:**

The thickness of the labial gingiva was increased about 0.42 to 2.00 mm after PhMT-s. At the end of pre-orthognathic surgical orthodontic treatment, the thickness of the labial gingiva was increased about − 0.14 to 1.32 mm compared to the baseline and no gingival recession occurred after the pre-orthognathic surgical orthodontic treatment. The histologic results demonstrated that the grafts obtained from the PhMT-s site exhibited increased deposition of collagen fibers. Moreover, the proportion of type III collagen increased and the grafts displayed significantly reduced positive expression of CD31 and OCN.

**Conclusions:**

PhMT-s increased the thickness of the soft tissue, stabilizing the gingival margin for teeth exhibiting a thin periodontal phenotype and undergoing labial movement. This is attributed to the increased deposition of collagen fibers.

**Supplementary Information:**

The online version contains supplementary material available at 10.1186/s12903-024-04630-x.

## Introduction

Patients with skeletal angle Class III malocclusions usually have inadequate hard and soft tissue volume of the mandibular anterior teeth [1]. However, the objective of orthodontic treatment for these patients is decompensation of the mandibular anterior teeth. This means that the teeth will undergo buccal orthodontic proclination which may move the teeth outside the osseous boundaries and predispose the teeth to gingival recession, leading to hypersensitivity, caries development and aesthetic impairment [2].

Therefore, periodontal phenotype modification therapy (PhMT) is advised before orthodontic treatment, including hard tissue augmentation (PhMT-b) or soft tissue augmentation (PhMT-s), for these high-risk patients [3]. PhMT-b includes corticotomy-assisted orthodontic therapy (CAOT) combined with simultaneous bone augmentation which is also termed periodontally accelerated osteogenic orthodontics (PAOO) [4] or surgically facilitated orthodontic therapy (SFO) [5]. PhMT-s includes free gingival grafting for increasing keratinized gingival width and connective tissue grafting for increasing the gingival thickness [3].

According to a consensus report on the use of modified periodontal phenotypes in preparation for orthodontic and restorative treatment from the American Academy of Periodontology, experts have concluded that PhMT-b is beneficial for patients undergoing orthodontic treatments [6]. The benefits include accelerating tooth movement, increasing the stability of orthodontic outcomes, expanding the boundaries for orthodontic treatments and reducing the incidence of periodontal complications. However, these procedures may lead to some potential risks such as root damage, gingival papilla defects and pulpal devitalization. Moreover, the reported results of PhMT-b indicated that the amount of bone gain at the root cervical region was low, and most bone gain was obtained in the apical region [7]. This finding could be explained by invasion of the gingival soft tissue, exclusion of some bone substitutes from the gingival margin and downward movement of other bone substitutes due to gravity. Moreover, the experts concluded that if the thickness of the coronal third of the periodontal soft tissue is less than 1 mm, the gingival phenotype is defined as a thin phenotype, which is prone to gingival recession [8]. Therefore, it is recommended to perform PhMT-s in advance of PhMT-b for patients with a generalized thin gingival phenotype to stabilize the gingival margin and optimize the augmentation outcome [9].

PhMT-s is extensively utilized around dental implants, effectively altering the peri-implant soft tissue phenotype from thin to thick, ultimately minimizing the occurrence of mucosal recession around implants [10]. Despite its widespread application, however, limited research has been conducted to determine whether PhMT-s can effectively prevent gingival recession among patients suffering from skeletal angle Class III malocclusion with a thin periodontal phenotype undergoing labial proclination. The labial proclination of these teeth often results in gingival recession. Our study of four cases demonstrated that the use of soft tissue grafts as a means to modify the thin periodontal phenotype can effectively prevent gingival recession in these patients.

## Materials and methods

### Inclusion criteria

The inclusion criteria for our study were as follows: patients who were diagnosed with skeletal angle Class III malocclusion necessitating labial proclination of the lower anterior teeth. Additionally, these patients were required to display a thin periodontal phenotype and alveolar bone loss at the root cervical region, without periodontal pockets resulting from bone dehiscence. Furthermore, patients exhibiting mild gingival recession and a thin periodontal phenotype were also eligible. Candidates must maintain excellent oral hygiene, defined as a full-mouth plaque score below 20%, and possess healthy periodontal tissue or well-controlled inflammation, characterized by fewer than 10% bleeding sites and probing depths not exceeding 3 mm, as well as keratinized gingiva dimensions of at least 2 mm. Finally, patients without a history of periodontal surgery in the past six months and nonsmokers were considered eligible for inclusion in the study.

### Common clinical procedures

Four patients, who had inadequate hard and soft tissue volume at the mandibular anterior teeth, were diagnosed with Skeletal Class III malocclusion, and received surgical-orthodontic treatment. The patients were treated with PhMT-s at the mandibular anterior teeth prior to orthodontic movement. All patients signed informed consent forms. Table 1 describes the age, sex, L1-MP (pre-treatment and post-orthodontic treatment), advancement of the mandibular incisors at the cervical level and advancement of the mandibular incisor tip after orthodontic treatment (Table 1).

**Table 1:** Basic information of the four patients

Patient	Age	Sex	L1-MP (Pre-treatment)	L1-MP (Post-orthodontic treatment)	CA				TA			
Tooth #					42	41	31	32	42	41	31	32
1	22	Female	62.5°	75.4°	1.24	1.84	1.85	1.17	3.29	4.07	5.51	3.94
2	24	Male	64.8°	75.6°	0.56	0.43	1.37	0.89	1.59	1.94	1.37	2.05
3	23	Male	77.8°	85.2°	0.54	0.95	1.21	1.17	1.31	3.29	3.43	1.83
4	32	Female	75.6°	79.3°	1.11	0.74	0.07	0.74	1.11	1.45	1.71	2.03

CA: Advancement of the mandibular incisor at the cervical level

TA: Advancement of the mandibular incisor tip

### Orthodontic examination

A medical and dental history were obtained for the patients, followed by a thorough facial and dental examination. Pretreatment cephalometric radiographs and analysis were required to determine the skeletal class. The SNA, SNB, ANB, and LI-MP points and angles were measured and compared with those measured via lateral cephalometry before orthognathic surgery (at the end of the pre-surgical orthodontic treatment). The mandibular incisors were planned to undergo proclination. This process would reposition their roots out of the osseous envelope of the alveolar process, resulting in a greater tendency for developing gingival recession.

### Periodontal examination

**Fig. 1:**
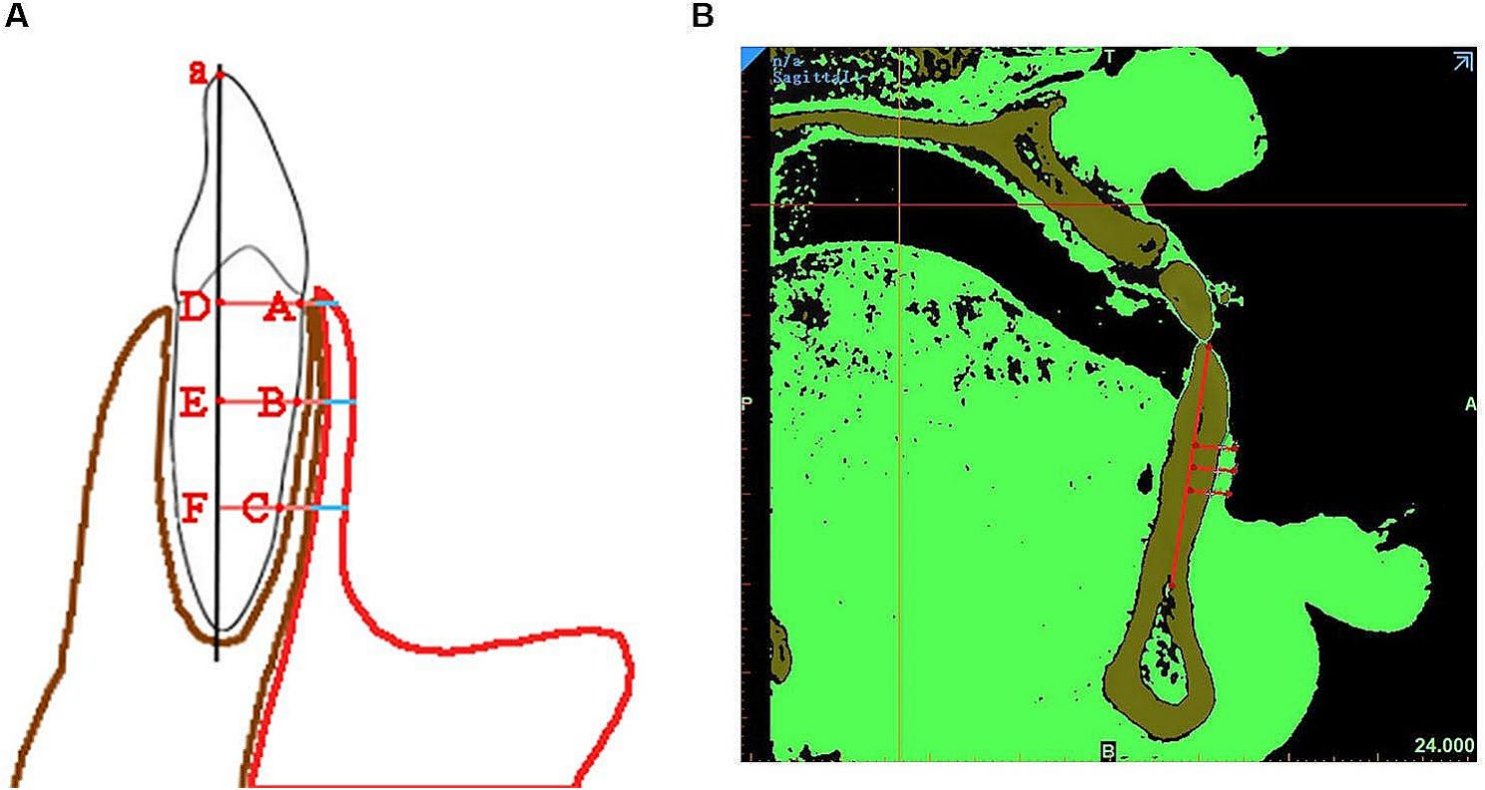
The measurement of the labial gingival thickness on the cross-sectional CBCT images with Mimics software. **A** The pattern diagram of the measurement of the labial gingival thickness. Point A, B, C presented the CEJ, 3 mm apical to the CEJ and 6 mm apical to the CEJ. Line a presented the long axis of the measured tooth. Then, we drew three lines perpendicular to line a through point A, B, C and got Line AD, BE, CF. Finally, measure the thickness of the labial gingiva on the Line AD, BE, CF. **B** The gingival thickness was measured at the level of the CEJ and at distances of 3 mm and 6 mm apical to the CEJ

The periodontal phenotype of the mandibular incisors was evaluated as described by Kan et al. [10] Meanwhile, CBCT was used to evaluate the thickness of the gingiva at the labial aspects of the incisors at three time points (baseline, in advance of the tooth movement and after tooth decompensation). A high-resolution (voxel size = 0.125 mm) CBCT image was taken with a KODAK 9500 device (Carestream Health, Canada) set at 120 kV and 10 mA. A self-retainable retractor was placed to separate the lip and the gingiva. The CT scans were limited to a 3 × 3 cm field of view (FOV), and the exposure time was 10.8 s. The CT data were imported into Mimics (version 12.1, Materialise Medical Co, Leuven, Belgium). Two adequate segmentation masks were created for the hard tissue and the soft tissue. The threshold of the hard tissue was 226 HU-3071 HU, and enamel, cementum, dentin, and alveolar bone were included. The threshold of the soft tissue was − 700 HU-3071 HU, and the gingiva, enamel, cementum, dentin, and alveolar bone were included. Then the anterior mandibular incisors were brought into focus on a cross-sectional image. The gingival thickness (GT) was measured along the long axis of the measured tooth. The gingival thickness was estimated at the level of the cementoenamel junction (CEJ) (GT0), the distance of 3 mm apical to the CEJ (GT3) and the distance of 6 mm apical to the CEJ (GT6) (Fig. 1). Two inspectors (Zhixu Liu and Mengdi Li) each measured the thickness twice, and the average of the measurements was regarded as the result (Supplementary Table 1, Supplementary Table 2, Supplementary Table 3).

### Periodontal phenotype modification therapy with soft tissue augmentation (PhMT-s)

The patients were treated with minimally invasive vestibular incision subperiosteal tunnel access (VISTA) combined with autogenous connective tissue grafts. The procedures were all performed by the same periodontist (Jing Ni). The methods were as follows: After administering local infiltration anesthesia, three vertical vestibular incisions of sufficient length were made in the mandibular midline frenum and the distal sides of the right and left mandibular canines. A subperiosteal tunnel was elevated from the right canine’s distal side to the left canine’s distal side. It extended from the vestibule to the gingival margin. The tunnel was adequately released to fit a 2 mm thick and at least 5 mm apico-coronal-dimensioned autogenous connective tissue graft. Then, the autogenous connective tissue grafts employed a de-epithelialized technique were harvested from the two sides of the palate because the graft harvested from one side was not long enough. Prior to suturing, the apico-coronal dimension and thickness of the autogenous connective tissue graft were precisely measured using a manual probe. An autogenous connective tissue graft was inserted from the incision to the tunnel guided by the periodontal probe and was stabilized at the CEJ of the tooth with 5.0 polyglactin 910 sutures. Then, the grafts were covered with a coronally advanced flap with 5.0 polyglactin 910 sling sutures. The three vertical incisions were closed with 6.0 polypropylene sutures. Primary closure of the palate area was achieved with 4.0 polyglactin 910 sutures. The sutures were removed 2 weeks after the surgery. Patients were treated with antibiotics and ibuprofen after surgery, and a 0.12% chlorhexidine rinse was performed three times a day for 2 weeks.

### Histologic evaluation

One patient expressed dissatisfaction with the excessive thickening on the labial side of their lower anterior teeth, which compromised their aesthetics. Therefore, a decision was made to proceed with a second surgical intervention aimed at restoring the gingival contour to its desired aesthetics. Following the elevation of the gingival flap, histological samples were collected, both from beneath the soft tissue flap and from the surface of the alveolar bone, as depicted in Figure S1. A small part of the tip of the tissue graft was harvested from the palate of the other patients for comparison. Paraformaldehyde-fixed samples were paraffin-embedded, and 5-µm-thick sections were cut and subjected to hematoxylin and eosin (H&E), Masson, and Sirius Red staining according to standard protocols. For immunohistochemistry, the primary antibodies included anti-rabbit CD31 (Servicebio, Wuhan, China) and anti-rabbit OCN (Servicebio, Wuhan, China). HRP-conjugated goat anti-rabbit IgG was used as the secondary antibody and visualized by 3,3’-diaminobenzidine (Servicebio, Wuhan, China). All images were captured by a digital slide scanner (Pannoramic 250/midi, 3DHISTECH, Hungary), while the Sirius Red-stained sections were photographed under polarized light.

## Results

### Case report 1

**Fig. 2:**
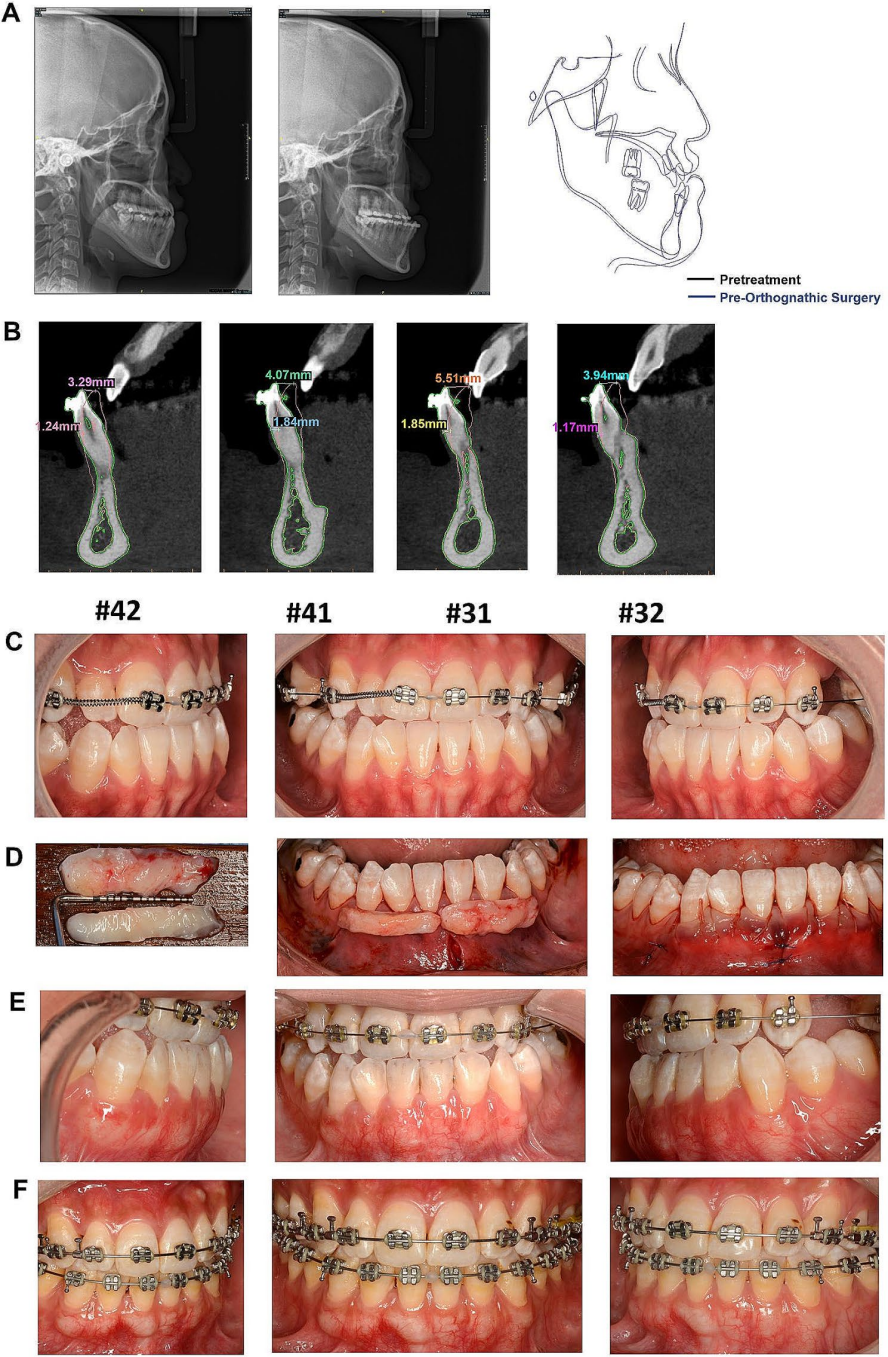
Cephalometric radiographs, CBCT and clinical photographs of case 1. **A** The comparison of the cephalometric radiographs of the baseline and the end of pre orthognathic surgical orthodontic treatment. **B** The superimposition images of the #31#32#41#42 CBCT data at baseline and at the end of pre orthognathic surgical orthodontic treatment. **C** The appearance of the anterior mandibular teeth with thin periodontal phenotype at baseline. **D** Subperiosteal tunnel was extended from the vestibular to the gingival margin, which accommodated a 2 mm thick and at least 5 mm apico-coronal-dimensioned autogenous connective tissue graft. **E** The photographs at three months after the PhMT-s surgery. **F** The photographs at the end of orthognathic surgery

A 22-year-old woman was referred to a periodontist for PhMT-s. Pretreatment cephalometric radiography and analysis revealed severe skeletal Class III (ANB angle − 2.4°, Wits Appraisal − 7.2 mm). The retroclination mandibular incisors (L1-MP 62.5°) needed to be upright for the orthognathic procedure, and the teeth were placed relative to their supporting bone. PhMT-s was applied before orthodontic treatment. The thickness of the labial gingiva at the surgical site had increased substantially three months after treatment without any complications, and the increase in the thickness of the labial gingiva was about 0.9 to 1.8 mm. The orthodontic treatment began three months post-PhMT-s. At the end of pre-orthognathic surgical orthodontic treatment, the thickness of the labial gingiva was increased about 0.36 to 1.19 mm compared to the baseline. Moreover, the L1-MP angle increased to 75.4° and the advancement of the mandibular incisor tip was approximately 3 to 5 mm (Fig. 2; Tables 1 and 2). Concurrently, this patient displayed gingival recession ranging from 0.5 to 1 mm in teeth #41, #42, #31, and #32. Following the soft tissue augmentation surgery, a notable increase in gingival height was observed. Upon completion of the orthodontic treatment, the exposed root surface was fully covered, and the gingival height remained stable (Fig. 2).

**Table 2:** Changes in gingival thickness for patient 1

Tooth#	Patient 1					
	Changes in gingival thickness of phase 1 (pre-orthodontic movement after PhMT-s compared to baseline)			Changes in gingival thickness of phase 2 (completion of tooth decompensation compared to baseline)		
	GT0	GT3	GT6	GT0	GT3	GT6
42	1.4	1.4	0.9	0.47	0.67	0.7
41	1.3	1.8	1.5	0.66	1.19	0.82
31	0.9	1.2	1.1	0.36	0.54	0.75
32	1.1	1.1	1.3	0.5	1.03	1.03

Changes in gingival thickness of phase 1: Changes in gingival thickness after treated with PhMT-s at the mandibular anterior teeth in advance of the orthodontic movement compared to baseline (Pre-PhMT-s treatment)

Changes in gingival thickness of phase 2: Changes in gingival thickness at the completion of tooth decompensation compared to baseline (Pre-PhMT-s treatment)

### Case report 2

**Fig. 3:**
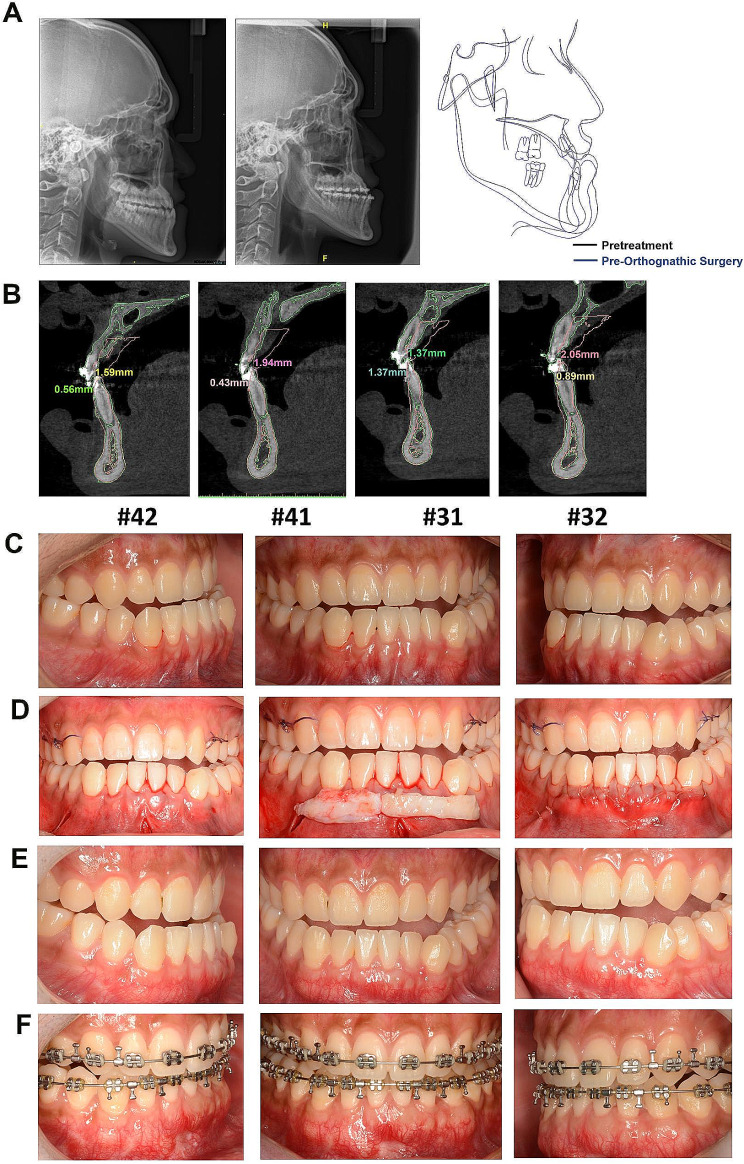
Cephalometric radiographs, CBCT and clinical photographs of case 2. **A** The comparison of the cephalometric radiographs of the baseline and the end of pre orthognathic surgical orthodontic treatment. **B** The superimposition images of the #31#32#41#42 CBCT data at baseline and at the end of pre orthognathic surgical orthodontic treatment. **C** The appearance of the anterior mandibular teeth with thin periodontal phenotype at baseline. **D** Subperiosteal tunnel was extended from the vestibular to the gingival margin, which accommodated a 2 mm thick and at least 5 mm apico-coronal-dimensioned autogenous connective tissue graft. **E** The photographs at three months after the PhMT-s surgery. **F** The photographs at the end of orthognathic surgery

A 24-year-old man with a thin periodontal phenotype was advised to undergo the PhMT-s procedure. He was diagnosed with skeletal Class III malocclusion (ANB angle − 4.1°, Wits Appraisal − 10.5 mm). The L1-MP angle of 64.8° reflected a retroclination mandibular incisors. After the PhMT-s, no complications were reported by the patient. L1-MP was 75.6° after pre-surgical orthodontic treatment and the advancement of the mandibular incisor tip was approximately 1.5–2.05 mm. The gingival thickness of the mandibular teeth was improved approximately 0.42 to 1.94 mm after the PhMT-s procedure. At the end of pre-orthognathic surgical orthodontic treatment, the thickness of the labial gingiva had increased by approximately − 0.14 to 1.14 mm compared to that at baseline, and no gingival recession occurred after the pre-orthognathic surgical orthodontic treatment (Fig. 3; Tables 1 and 3).

**Table 3:** Changes in gingival thickness for patient 2

Tooth#	Patient 2					
	Changes in gingival thickness of phase 1 (pre-orthodontic movement after PhMT-s compared to baseline)			Changes in gingival thickness of phase 2 (completion of tooth decompensation compared to baseline)		
	GT0	GT3	GT6	GT0	GT3	GT6
42	0.42	1.58	0.49	0.06	0.74	0.03
41	0.8	1.94	1.62	0.28	0.52	1.02
31	0.55	1.56	1.32	0.37	1.14	0.54
32	1.41	1.68	1.67	0.88	1.05	−0.14

Changes in gingival thickness of phase 1: Changes in gingival thickness after treated with PhMT-s at the mandibular anterior teeth in advance of the orthodontic movement compared to baseline (Pre-PhMT-s treatment)

Changes in gingival thickness of phase 2: Changes in gingival thickness at the completion of tooth decompensation compared to baseline (Pre-PhMT-s treatment)

### Case report 3

**Fig. 4:**
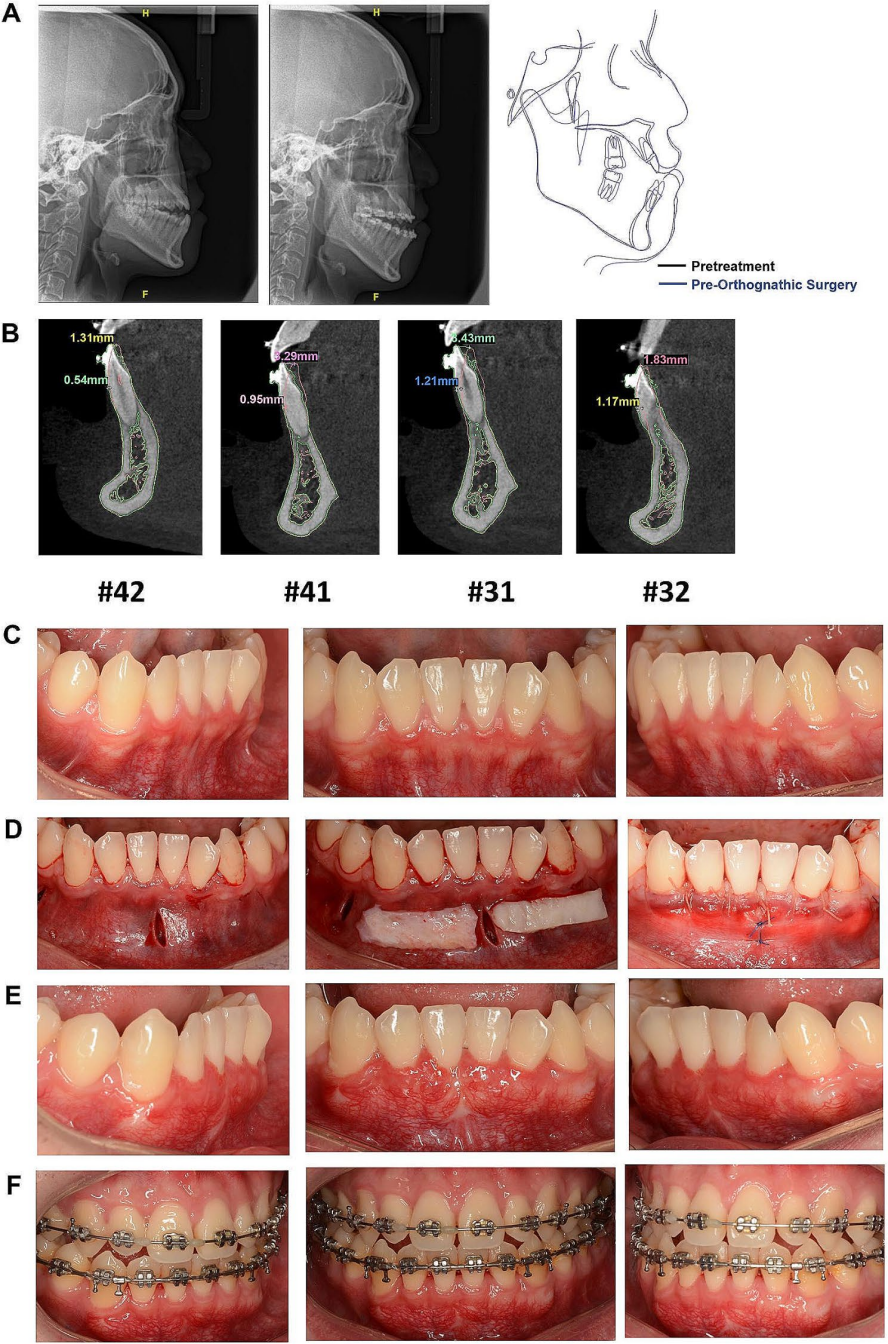
Cephalometric radiographs, CBCT and clinical photographs of case 3. **A** The comparison of the cephalometric radiographs of the baseline and the end of pre orthognathic surgical orthodontic treatment. **B** The superimposition images of the #31#32#41#42 CBCT data at baseline and at the end of pre orthognathic surgical orthodontic treatment. **C** The appearance of the anterior mandibular teeth with thin periodontal phenotype at baseline. **D** Subperiosteal tunnel was extended from the vestibular to the gingival margin, which accommodated a 2 mm thick and at least 5 mm apico-coronal-dimensioned autogenous connective tissue graft. **E** The photographs at three months after the PhMT-s surgery **F** The photographs at the end of orthognathic surgery

A 23-year-old man was referred to a periodontist for gingival phenotype evaluation. He was classified as having a thin periodontal phenotype and skeletal Class III malocclusion (ANB angle − 0.4°, Wits Appraisal − 8.1 mm). The treatment plan for the patient was to correct his skeletal class III malocclusion via orthognathic surgery. The mandibular incisors showed significant retroclination (L1-MP 77.8°). PhMT-s was suggested since his lower incisors would proclinate during the orthodontic treatment. The procedures were performed as described in the methods section. No complications were observed after the periodontic treatment. The thickness of the labial gingiva increased by approximately 0.59 to 2.00 mm after PhMT-s. At the end of the pre-orthognathic surgical orthodontic treatment, the thickness of the labial gingiva increased by approximately − 0.05 to 1.32 mm compared to the baseline. No gingival recession was observed at the termination of the orthodontic treatment. Table 4 describes the differences in the gingival thickness of the labial mandibular teeth. At the end of the pre-orthognathic-surgical orthodontic treatment, L1-MP turned to 85.2° and the advancement of the mandibular incisor tip was approximately 1.3 to 3.4 mm (Fig. 4; Tables 1 and 4).

**Table 4:** Changes in gingival thickness for patient 3

Tooth#	Patient 3					
	Changes in gingival thickness of phase 1 (pre-orthodontic movement after PhMT-s compared to baseline)			Changes in gingival thickness of phase 2 (completion of tooth decompensation compared to baseline)		
	GT0	GT3	GT6	GT0	GT3	GT6
42	0.59	0.59	2	0.03	0.51	−0.05
41	0.73	1.59	1.54	0.1	1	0.47
31	0.71	1.64	1.58	0.41	1.32	0.84
32	0.7	1.3	1.09	0.15	0.36	0.3

Changes in gingival thickness of phase 1: Changes in gingival thickness after treated with PhMT-s at the mandibular anterior teeth in advance of the orthodontic movement compared to baseline (Pre-PhMT-s treatment)

Changes in gingival thickness of phase 2: Changes in gingival thickness at the completion of tooth decompensation compared to baseline (Pre-PhMT-s treatment)

### Case report 4

**Fig. 5:**
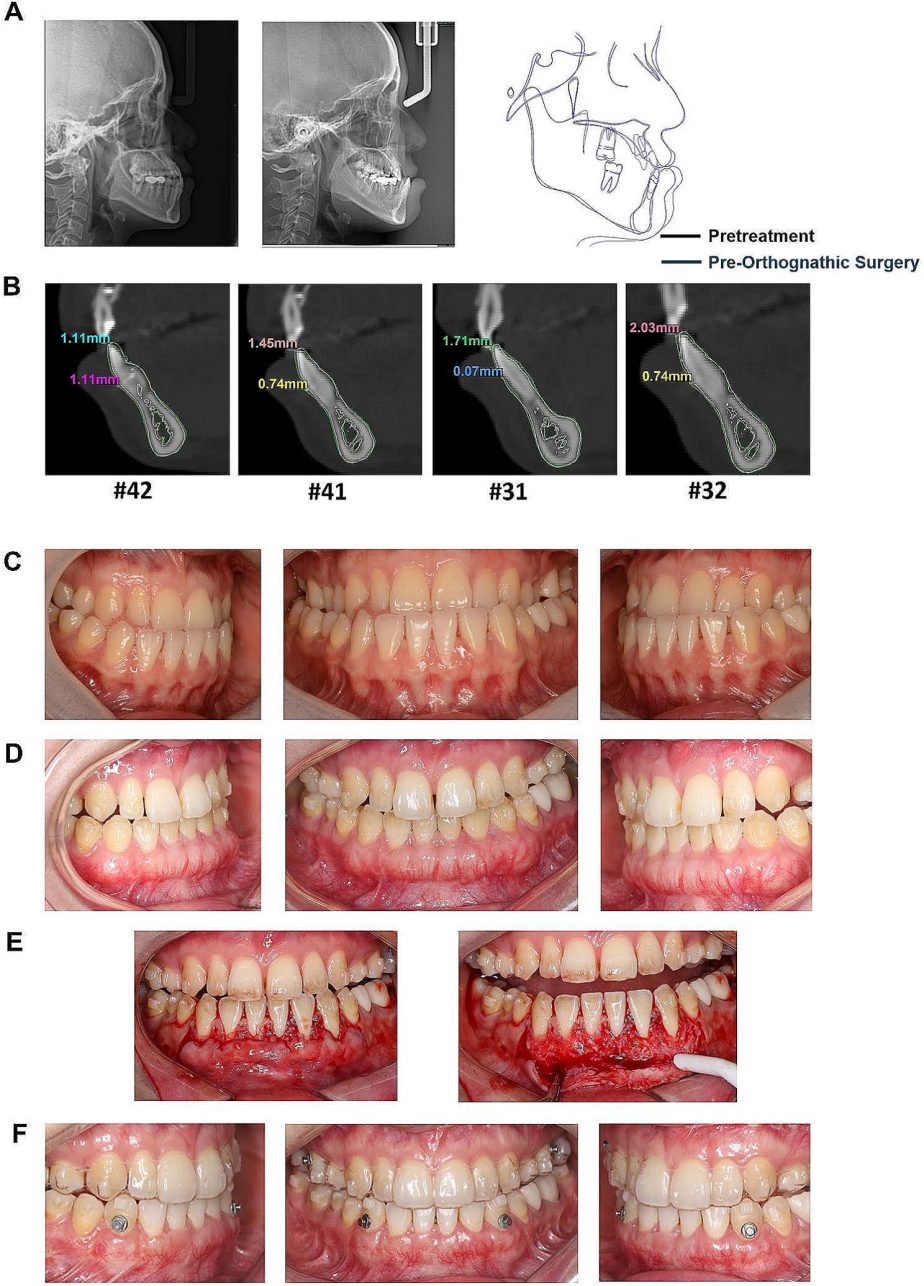
Cephalometric radiographs, CBCT and clinical photographs of case 4. **A** The comparison of the cephalometric radiographs of the baseline and the end of pre orthognathic surgical orthodontic treatment. **B** The superimposition images of the #31#32#41#42 CBCT data at baseline and at the end of pre orthognathic surgical orthodontic treatment. **C** The appearance of the anterior mandibular teeth with thin periodontal phenotype at baseline. **D** The photographs at the end of orthognathic surgical treatment. **E** A second surgical procedure aimed at restoring the gingival contour to its desired shape were conducted. Biopsies were obtained from beneath the soft tissue flap and from the exact surface of the alveolar bone. **F** The photographs at three months after the surgery aimed at restoring the gingival contour

A 32-year-old woman with a thin periodontal phenotype was advised to undergo the PhMT-s procedure. She was diagnosed with skeletal Class III malocclusion (ANB angle − 3.3°, Wits Appraisal − 10.5 mm). The L1-MP angle of 75.6° reflected a retroclination of the mandibular incisors. After the PhMT-s, no complications were reported by the patient. L1-MP was 79.3° after pre-surgical orthodontic treatment and the advancement of the mandibular incisor tip was approximately 1.1–2.0 mm (Table 1). Concurrently, this patient displayed gingival recession ranging from 0.5 to 2 mm in teeth #41, #42, #31, and #32. Following the soft tissue augmentation surgery, a notable increase in gingival height was observed. Upon completion of the orthodontic treatment, the exposed root surface was fully covered, and the gingival height remained stable. But the patient was not satisfied with the excessive thickening of the labial side of the lower anterior teeth after the orthognathic surgery. A second surgical procedure aimed at restoring the gingival contour to its desired shape was conducted. Biopsies were obtained from beneath the soft tissue flap and from the surface of the alveolar bone (Fig. 5).

**Fig. 6:**
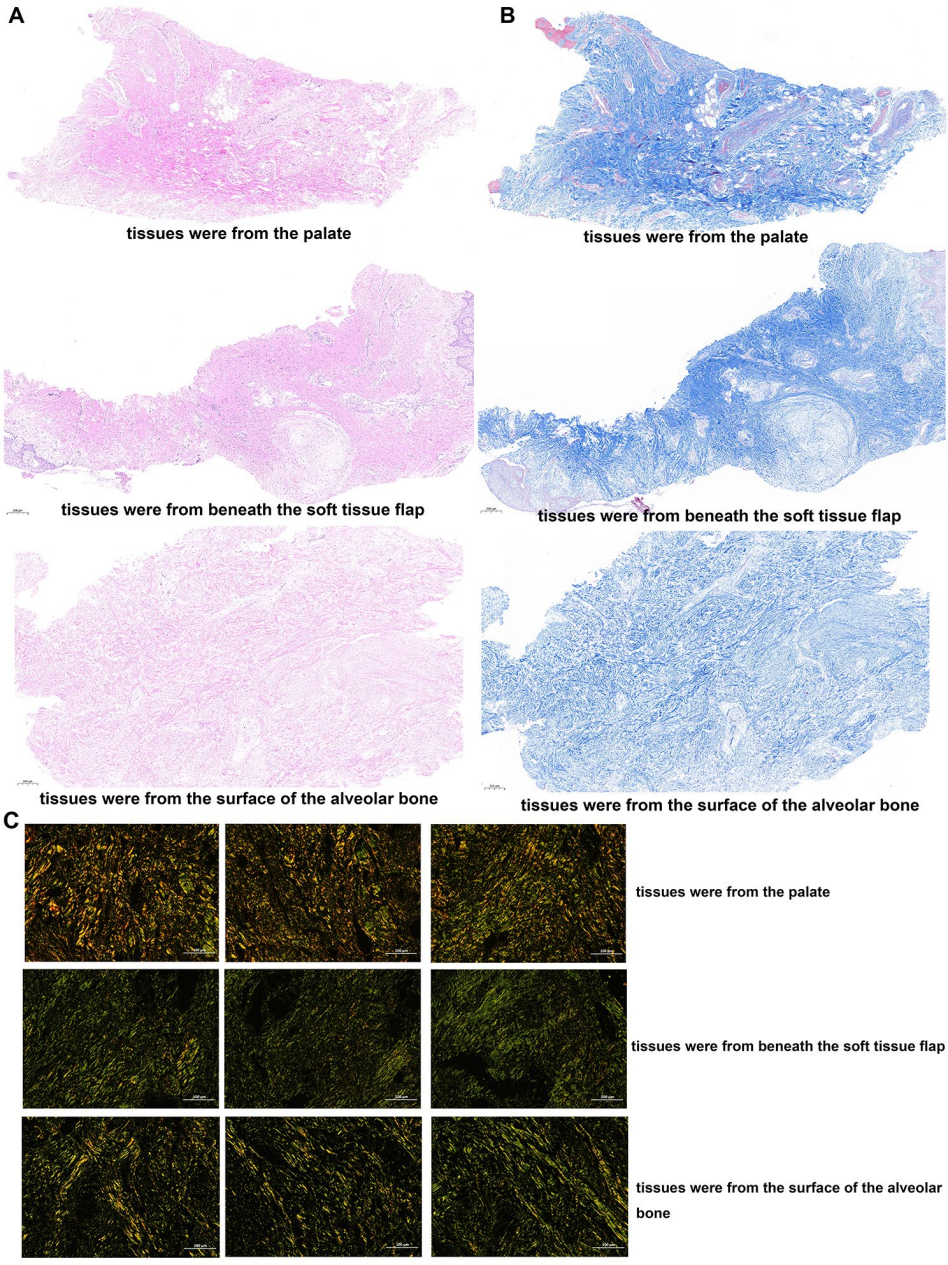
The results of hematoxylin and eosin H&E, Masson’s trichrome MT and Sirius Red staining. **A** The gingival connective tissue at the soft tissue augmentation site that develops following the transplantation of the palatal graft exhibits a higher density compared to the palatal subepithelial connective tissue. On the contrary, the palatal subepithelial connective tissue is richer in blood vessels than the gingival connective tissue that arises from the palatal graft transplantation. Notably, neither of these tissues demonstrates the presence of inflammatory cells. **B** Upon examination of sections stained with Masson’s trichrome the soft tissue formed following the transplantation of the palatal graft exhibited an increase in collagen fiber deposition and a corresponding decrease in angiogenesis. Furthermore, a distinct difference was apparent between the tissues sampled from beneath the soft tissue flap and those obtained from the surface of the alveolar bone. Tissue harvested from the alveolar bone surface exhibited a higher density of collagen fibers, greater homogeneity in fiber arrangement, and reduced vascularity. **C** Morphological differences between type I and type III collagen were analyzed through Sirius Red staining. We noted alterations in the collagen content, specifically in the subepithelial connective tissue graft from the palate and the soft tissue formed following the transplantation of the palatal graft. In the subepithelial connective tissue graft from the palate, type I and type III collagen were intertwined, with type I collagen being the dominant component surrounded by a minor amount of green-tinged type III collagen. Conversely, in tissues collected from beneath the soft tissue flap, type III collagen was predominant, encircled by a minor quantity of type I collagen exhibiting an orange hue. Notably, the proportion of type I and type III collagen in tissues harvested from the surface of the alveolar bone was approximately equal

**Fig. 7:**
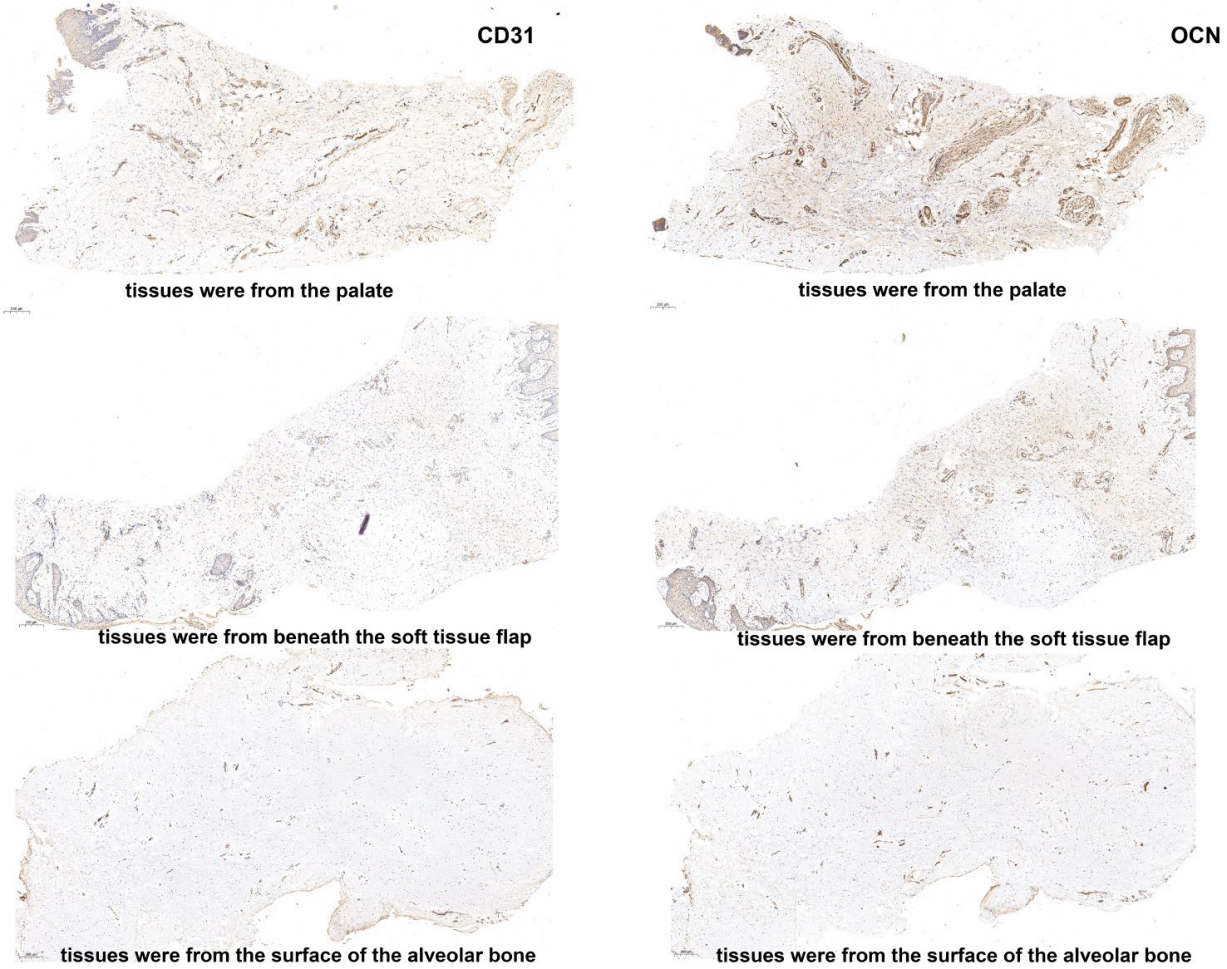
The immunochemistry of CD31 and OCN. The expression levels of CD31and OCN were significantly elevated in the subepithelial connective tissue graft from the palate compared to the soft tissue formed following the transplantation of the palatal graft

The histologic results demonstrated that, compared to grafts harvested from the palate, grafts obtained from the soft tissue augmentation site exhibited an increased deposition of collagen fibers along with a diminished angiogenic response. Furthermore, notable variations in the content of both type I and type III collagen were observed between the grafts from the two sites. Specifically, grafts from the palate were characterized by a preponderance of type I collagen, which was surrounded by a limited amount of green-tinged type III collagen. Conversely, grafts harvested from beneath the soft tissue flap exhibited a predominance of type III collagen, encircled by a small quantity of type I collagen exhibiting an orange hue. Notably, the proportions of type I and type III collagen were approximately equal in the tissues collected from the surface of the alveolar bone. These observations suggest that the collagen composition is altered in the soft tissue that forms following the insertion of the palatal graft (Fig. 6). Additionally, we examined the angiogenic and osteogenic potential of grafts from both sites. Notably, the grafts from the soft tissue augmentation site exhibited significantly lower positive expression of CD3 and OCN than did the grafts from the palate, indicating inferior angiogenic and osteogenic capacity (Fig. 7).

## Discussion

To our knowledge, this is the first case series study to verify the clinical effects of PhMT-s for patients with a thin periodontal phenotype and bone dehiscence who underwent Class III decompensation by comparing the labial gingival thickness at baseline, three months after PhMT-s (in advance of tooth movement) and after tooth decompensation. We found that the thickness of the labial gingiva was greater after PhMT-s and after tooth decompensation than at baseline. There was no gingival recession and root surface exposure after the orthodontic treatment. After soft tissue augmentation and the combined orthodontic treatment, the gingival height in the baseline recession area increased in two patients, resulting in an improvement in gingival recession. In regions of soft tissue thickening, the degree of gingival keratinization gradually increases, which can cause aesthetic problems. The histologic results demonstrated that grafts obtained from the soft tissue augmentation site exhibited increased deposition of collagen fibers and an increased proportion of type III collagen.

The incidence of bone dehiscence and gingival recession is greater for teeth with a thin periodontal phenotype exposed to orthodontic forces, such as arch expansion and labial proclination. In 2001, Wilcko et al. proposed a surgical technique that combines cortical osteotomy with bone substitute grafting to accelerate the orthodontic movement of teeth [11]. Moreover, this technique can increase the thickness of the buccal bone and maintain the stability of periodontal tissue. However, the tissue gain observed in the root cervical region remains questionable, as it closely resembles the findings associated with the augmentation of bony dehiscence around oral implants utilizing guided bone regeneration (GBR) techniques. Temmerman et al. conducted an RCT study to compare the clinical effects of bovine-derived xenografts in combination with autogenous bone chips versus those of xenografts alone for the augmentation of bony dehiscence around dental implants [12]. In both groups, the increase in thickness at the shoulder of the implant seemed to disappear after the healing phase. Mir-Mari and coworkers investigated the volume stability of the augmented region during suturing of the mucosal flap [13]. They found that the displacement of the bone substitutes predominately occurred at the shoulder of the implant during suturing. Therefore, some scholars have attempted to conduct soft tissue grafting around dental implants for contour augmentation, especially for immediate implants. Fujita et al. compared the horizontal dimensional changes of the facial bone and soft tissue following immediate implant placement with simultaneous soft tissue grafting or GBR for contour augmentation [14]. The authors concluded that CTG should be performed with immediate implant placement in cases where the preoperative mucosal contours need to be maintained. However, until now, no study has compared the clinical effects of soft tissue grafting and hard tissue augmentation for teeth undergoing orthodontic labial movements. The clinical data provided by this study could serve as a reference for future clinical studies.

In this pilot case series study, in Cases1, 2, and 3 the gingival thickness at the labial aspects of the mandibular anterior teeth was assessed across three distinct time points: baseline, prior to tooth movement, and following tooth decompensation. The measurement regions, CEJ-GT0, 3 mm apical to CEJ-GT3, and 6 mm apical to CEJ-GT6, are dependent on the impedance center of the tooth which is close to the rotation center for the proclination movement. When the lower anterior teeth undergo proclination, the soft and hard tissues on the labial side above the impedance center are subjected to pressure, leading to absorption and destruction. The aim of soft tissue augmentation is to increase the thickness of the compressed tissue side. The findings revealed that, despite the labial proclination of the teeth, the labial soft tissue thickness above the impedance center exhibited an increase compared to the baseline in most sites, attributable to the soft tissue augmentation surgery. However, solely at the two GT6 sites, negative values emerged, likely due to the 5 mm CTG width which aimed to minimize potential damage to the palatal vascular nerve bundle, thereby resulting in suboptimal augmentation outcomes at these specific locations. Meanwhile, it is imperative to emphasize that the variation in the thickness of the compressed tissue side is influenced by both the magnitude of the soft tissue augmentation and the degree of labial proclination of the teeth. To more accurately assess the influence of orthodontic movement on the thickness of gingival soft tissue, it is crucial to increase the sample size in future studies. Moreover, after the soft tissue augmentation and combined orthodontic treatment, the gingival height in the baseline recession area increased in two patients, resulting in an improvement in gingival recession. Numerous studies have confirmed that CTGs have a significantly better relative root coverage percentage than guided tissue regeneration [15, 16]. This indicates that for patients exhibiting minimal gingival recession and a thin periodontal phenotype in the mandibular anterior teeth, the need for labial proclination necessitates the utilization of soft tissue augmentation, which could refine the labial tissue contour and restore the gingival tissue to its desired height. Three patients in this study had bone dehiscence, and there was a lack of alveolar bone on the labial side of the root cervical region. There was no change in the thickness of the alveolar bone before and after treatment.

In Patient 4, histopathological analysis of the surgical area for gingival soft tissue augmentation demonstrated a substantial increase in collagen fiber deposition, which resulted in a denser and more stable labial soft tissue. This finding provides an explanation for the absence of gingival recession at the labial aspects of the mandibular anterior teeth with a thin periodontal phenotype undergoing labial proclination observed in Cases 1, 2, and 3. Interestingly, the proportions of type I and type III collagen varied in tissue samples collected from distinct regions, including the palate, beneath the soft tissue flap, and at the surface of the alveolar bone. This alteration could be attributed to shifts in the tissue microenvironment of gingival fibroblasts, ultimately leading to differential collagen secretory capabilities. Patricio et al. illustrated that the behavior of fibroblasts in the periodontal tissue microenvironment is influenced by a range of characteristics of the surrounding collagen fibers, which play a pivotal role in collagen synthesis and fiber reconstruction [17].

Previously, free gingival grafts (FGGs) and modified apically repositioned flaps were widely used for gingival augmentation to increase the keratinized tissue width. However, these procedures lead to poor color matching with neighboring tissues. In our study, the surgical technique that we chose was minimally invasive vestibular incision subperiosteal tunnel access (VISTA) combined with a connective tissue graft which has many benefits, including protecting the integrity of the papilla, leaving few postoperative scars and no bone exposure. Moreover, we employed a de-epithelialized technique to harvest the CTG, which consisted solely of the lamina propria. In the study conducted by Zucchelli et al. demonstrated that the use of the de-epithelialized technique resulted in a statistically significant and substantial increase in the thickness of the buccal gingiva, following recession coverage [18]. However, our observations revealed that in the regions where soft tissue thickening occurred, the degree of gingival keratinization gradually increased. This resulted in a slight color discrepancy compared to the neighboring tissues, potentially affecting the overall aesthetics. We hypothesize that this phenomenon may be attributed to isolated fragments of epithelium remaining within the graft following de-epithelialization. This is particularly relevant given the papillary interlocking between the epithelium and lamina propria [19]. Supporting this hypothesis, a study conducted by Harris demonstrated the presence of residual epithelium in 80% of grafts analyzed histologically after de-epithelialization [20]. Moreover, there are several disadvantages to connective tissue grafting, such as the morbidity and pain associated with the use of a second operating field and the limited dimensions of grafts harvested from the palate due to anatomical constraints [21–23]. In future studies, the clinical effects of connective tissue substitutes for the treatment of PhMT-s should be verified.

For future investigations involving patients with Class III decompensation, there are still some limitations regarding PhMT-s that need to be addressed: (1) Enhanced Long-Term Assessment: Integrating a rigorous long-term evaluation of PhMT-s efficacy will provide a more comprehensive view of the stability of soft tissue changes over extended periods. This approach is crucial for assessing the durability of treatment outcomes beyond the immediate post-treatment phase. (2) Expanding research scale: To further validate the observed results and enhance their generalizability, future research should prioritize expanding the study to include a larger and more diverse patient sample. This would allow for a more robust statistical analysis and strengthen the evidence supporting the efficacy of PhMT-s across various patient demographics and clinical contexts. (3) Exploration of Potential Drawbacks: It is imperative to acknowledge and explore potential drawbacks or limitations associated with PhMT-s. Factors such as donor site morbidity, patient discomfort, and variations in treatment outcomes among different periodontal phenotypes should be thoroughly investigated. Addressing these aspects will provide a more balanced assessment of the therapy’s overall utility in clinical practice.

## Conclusion

PhMT-s surgeries increase the thickness of the soft tissue, which could stabilize the gingival margin for teeth with a thin periodontal phenotype undergoing labial movement. A larger sample size should be studied to determine the difference in hard tissue augmentation (PhMT-b) or soft tissue augmentation (PhMT-s) in terms of maintaining periodontal health for patients with Class III decompensation undergoing labial proclination. Moreover, evaluating indicators such as patient-reported outcomes, treatment costs, postoperative swelling and pain should also be adopted to compare the two surgical techniques for advanced orthodontic labial movement.

### Electronic supplementary material

Below is the link to the electronic supplementary material.

Supplementary Material 1: Fig. 1. Clinical photographs of case 4.

Supplementary Material 2: Table S1. The thickness of the labial gingiva for Patient 1

Supplementary Material 3: Table S2. The thickness of the labial gingiva for Patient 2

Supplementary Material 4: Table S3. The thickness of the labial gingiva for Patient 3

## Data Availability

The data underlying this article will be shared on reasonable request from the corresponding author.

## Abbreviations

PhMT: Periodontal phenotype modification therapy
PhMT-b: Periodontal phenotype modification therapy with hard tissue augmentation
PhMT-s: Periodontal phenotype modification therapy with soft tissue augmentation
CAOT: Corticotomy-assisted orthodontic therapy
PAOO: Periodontally accelerated osteogenic orthodontics
SFO: Surgically facilitated orthodontic therapy
VISTA: Vestibular incision subperiosteal tunnel access
