# Supplementary material for: A preliminary investigation into the impact of soft tissue augmentation-based periodontal phenotype modification therapy for patients exhibiting class III decompensation
Source: BMC Oral Health. 2024 Aug 2;24:880. doi: 10.1186/s12903-024-04630-x (PMC11297605; doi:10.1186/s12903-024-04630-x)

**Supplementary Fig1. clinical photographs of case 4.**

A. The photographs at the end of orthognathic surgical treatment.

B. A second surgical procedure aimed at restoring the gingival contour to its desired shape were conducted. Biopsies were obtained from beneath the soft tissue flap and from the exact surface of the alveolar bone.

C. After surgical suturing, the photographs revealed the outcome of the procedure.

D. The photographs were captured during the removal of stitches.

E. The photographs at three months after the surgery aimed at restoring the gingival contour.


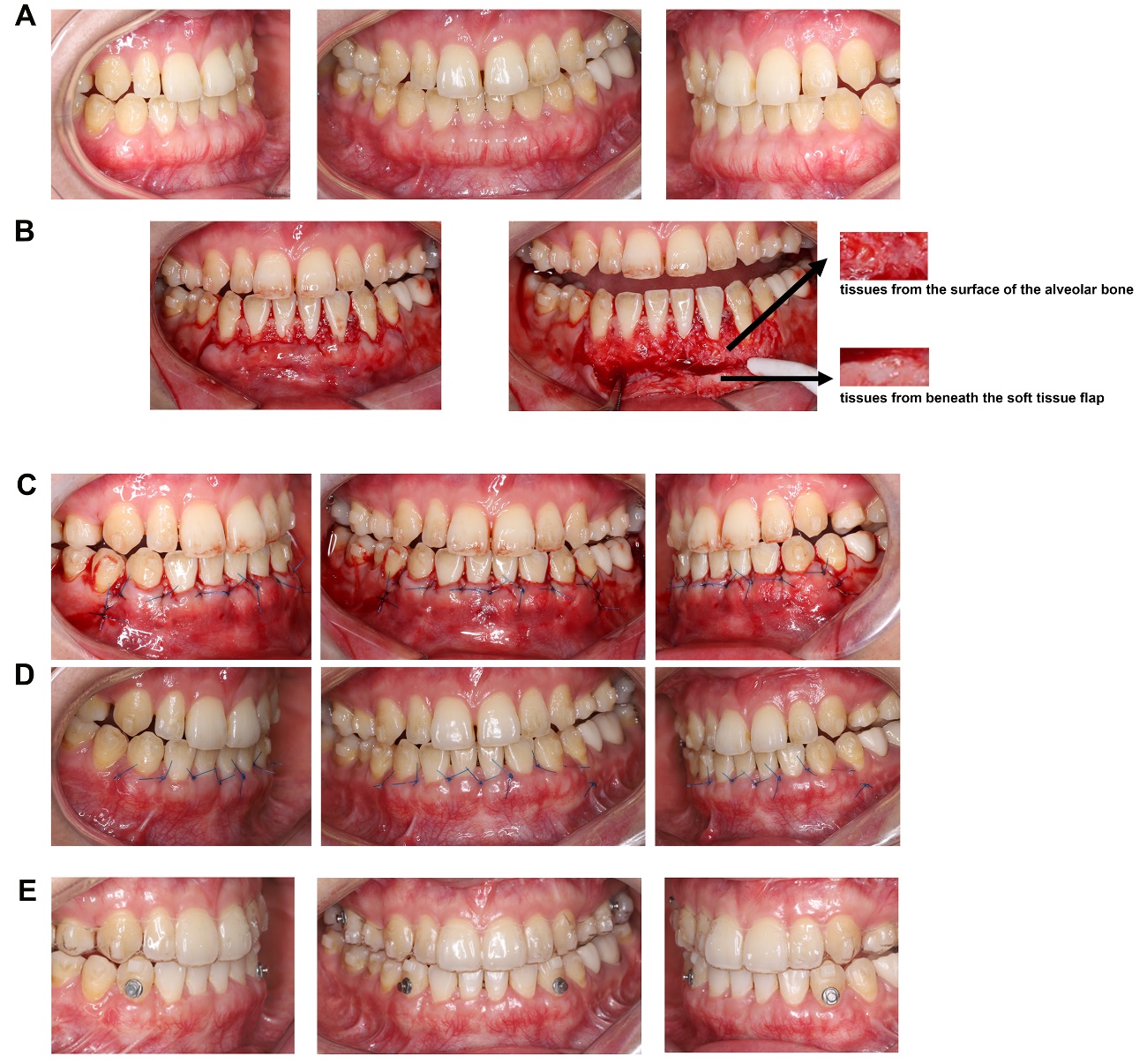

Supplement: Supplementary file 1 — Supplementary Material 1: Fig. 1. Clinical photographs of case 4. [file 12903_2024_4630_MOESM1_ESM.docx]
