# Supplementary material for: A preliminary investigation into the impact of soft tissue augmentation-based periodontal phenotype modification therapy for patients exhibiting class III decompensation
Source: BMC Oral Health. 2024 Aug 2;24:880. doi: 10.1186/s12903-024-04630-x (PMC11297605; doi:10.1186/s12903-024-04630-x)
Supplement: Supplementary file 2 — Supplementary Material 2: Table S1. The thickness of the labial gingiva for Patient 1 [file 12903_2024_4630_MOESM2_ESM.docx]

**Table S1. The thickness of the labial gingiva for Patient 1**

| Tooth# | Patient 1 | | | | | | | | | |
| --- | --- | --- | --- | --- | --- | --- | --- | --- | --- | --- |
|  | Pre-PhMT-s treatment  values | | | Pre-orthodontic treatment values | | | Pre-Orthognathic surgery values | | | |
|  | GT0 | GT3 | GT6 | GT0 | GT3 | GT6 | GT0 | GT3 | GT6 |  |
| 42 | 0.2 | 0.7 | 0.7 | 1.6 | 2.1 | 1.6 | 0.67 | 1.37 | 1.4 |  |
| 41 | 0 | 0 | 0 | 1.3 | 1.8 | 1.5 | 0.66 | 1.19 | 0.82 |  |
| 31 | 0.5 | 0.4 | 0.3 | 1.4 | 1.6 | 1.4 | 0.86 | 0.94 | 1.05 |  |
| 32 | 0.6 | 0.5 | 0.5 | 1.7 | 1.6 | 1.8 | 1.1 | 1.53 | 1.53 |  |

**GT0: the thickness of the labial gingiva at the CEJ**

**GT3: the thickness of the labial gingiva at a distance of 3 mm apical to the CEJ**

**GT6: the thickness of the labial gingiva at a distance of 6 mm apical to the CEJ**
