# Supplementary material for: A preliminary investigation into the impact of soft tissue augmentation-based periodontal phenotype modification therapy for patients exhibiting class III decompensation
Source: BMC Oral Health. 2024 Aug 2;24:880. doi: 10.1186/s12903-024-04630-x (PMC11297605; doi:10.1186/s12903-024-04630-x)
Supplement: Supplementary file 3 — Supplementary Material 3: Table S2. The thickness of the labial gingiva for Patient 2 [file 12903_2024_4630_MOESM3_ESM.docx]

**Table S2. The thickness of the labial gingiva for Patient 2**

| Tooth# | Patient 2 | | | | | | | | | |
| --- | --- | --- | --- | --- | --- | --- | --- | --- | --- | --- |
|  | Pre-PhMT-s treatment  values | | | Pre-orthodontic treatment values | | | Pre-Orthognathic surgery values | | | |
|  | GT0 | GT3 | GT6 | GT0 | GT3 | GT6 | GT0 | GT3 | GT6 |  |
| 42 | 0.87 | 0.76 | 2.17 | 1.29 | 2.34 | 2.66 | 0.93 | 1.5 | 2.2 |  |
| 41 | 0.67 | 0.43 | 0.64 | 1.47 | 2.37 | 2.26 | 0.95 | 0.95 | 1.66 |  |
| 31 | 1 | 0.36 | 0.87 | 1.55 | 1.92 | 2.19 | 1.37 | 1.5 | 1.41 |  |
| 32 | 0.62 | 0.45 | 1.31 | 2.03 | 2.13 | 2.98 | 1.5 | 1.5 | 1.17 |  |

**GT0: the thickness of the labial gingiva at the CEJ**

**GT3: the thickness of the labial gingiva at a distance of 3 mm apical to the CEJ**

**GT6: the thickness of the labial gingiva at a distance of 6 mm apical to the CEJ**
