# Supplementary material for: A preliminary investigation into the impact of soft tissue augmentation-based periodontal phenotype modification therapy for patients exhibiting class III decompensation
Source: BMC Oral Health. 2024 Aug 2;24:880. doi: 10.1186/s12903-024-04630-x (PMC11297605; doi:10.1186/s12903-024-04630-x)
Supplement: Supplementary file 4 — Supplementary Material 4: Table S3. The thickness of the labial gingiva for Patient 3 [file 12903_2024_4630_MOESM4_ESM.docx]

**Table S3. The thickness of the labial gingiva for Patient 3**

| Tooth# | Patient 3 | | | | | | | | | |
| --- | --- | --- | --- | --- | --- | --- | --- | --- | --- | --- |
|  | Pre-PhMT-s treatment  values | | | Pre-orthodontic treatment values | | | Pre-Orthognathic surgery values | | | |
|  | GT0 | GT3 | GT6 | GT0 | GT3 | GT6 | GT0 | GT3 | GT6 |  |
| 42 | 0.89 | 1.05 | 1.05 | 1.48 | 1.64 | 3.05 | 0.92 | 1.56 | 1 |  |
| 41 | 1.05 | 0.49 | 0.76 | 1.78 | 2.08 | 2.3 | 1.15 | 1.49 | 1.23 |  |
| 31 | 0.98 | 0.58 | 0.49 | 1.69 | 2.22 | 2.07 | 1.39 | 1.9 | 1.33 |  |
| 32 | 1.08 | 1.14 | 1.01 | 1.78 | 2.44 | 2.1 | 1.23 | 1.5 | 1.31 |  |

**GT0: the thickness of the labial gingiva at the CEJ**

**GT3: the thickness of the labial gingiva at a distance of 3 mm apical to the CEJ**

**GT6: the thickness of the labial gingiva at a distance of 6 mm apical to the CEJ**
